# Effects of Variable-Temperature Roasting on the Flavor Compounds of Xinjiang Tannur-Roasted Mutton

**DOI:** 10.3390/foods13193077

**Published:** 2024-09-27

**Authors:** Jian Wei, Li Wang, Xin Ma, Zequan Xu, Zirong Wang

**Affiliations:** 1 College of Life and Geographic Sciences, Kashi University, Kashi 844006, China; weijianfood@163.com (J.W.); 15294195001@163.com (L.W.); 2 College of Food Science and Pharmacy, Xinjiang Agricultural University, Urumqi 830052, China; maxin913@xjau.edu.cn (X.M.); wangzirong212@126.com (Z.W.)

**Keywords:** variable temperature, flavor compounds, roasted mutton, burning charcoal roasting, amino acids, free fatty acids

## Abstract

This study investigates the effect of variable-temperature roasting on the flavor compounds of Xinjiang tannur-roasted mutton. Gas chromatography coupled with ion mobility spectroscopy (GC-IMS) was used to compare and analyze the volatile components and flavor fingerprints of Xinjiang tannur-roasted mutton using variable-temperature electrically heated air roasting (VTR), constant-temperature electrically heated air roasting (EHAR), and constant-burning charcoal roasting (BCR) techniques. The changes in fatty acids and free amino acids in Xinjiang tannur-roasted mutton under different roasting conditions were compared. By using GC-IMS analysis, 11 flavor compounds, including 4-methyl-3-penten-2-one, isoamyl propionate, trans-2-heptenal, trans-2-heptenal, 2-hexanone, n-hexanol, 2-hexenal, 2-ethylfuran, and ethyl 2-methylbutanoate, were identified as characteristic volatile compounds in the temperature-controlled electrothermal roasting of Xinjiang tannur-roasted mutton using the following conditions: 0–4 min, 300 °C; 5–10 min, 220 °C; and 11–17 min, 130 °C (VTR_3_). Through principal component analysis, it was found that the substances with the highest positive correlation with PC1 and PC2 were n-hexanol and 3-methylbutanol. The sensory evaluation showed that VTR_3_ had high acceptability (*p* < 0.05) and a fat flavor (*p* < 0.05). There was no significant difference in the total fatty acid (TFA) content between the VTR_3_ and burning charcoal roast for 1–17 min at 300 °C (BCR_3_) (*p* > 0.05), but it was lower than that in the other experimental groups (*p* < 0.05). The lowest proportion of glutamic acid content in VTR_3_ was 22.44%, and the total free amino acid content in the electric thermostatic roasting for the 1–17 min, 300 °C (EHAR_3_) group (347.05 mg/100 g) was significantly higher than that in the other experimental groups (*p* < 0.05). By using Spearman correlation analysis, the roasting loss rate showed a highly significant negative correlation with essential amino acids (EAAs), non-essential amino acids (NEAAs), and total free amino acids (TAAs) (the correlation coefficients (*r*) were 0.82, 0.87, and 0.87, respectively) with *p* < 0.01. There was no correlation between changes in the free amino acid content and fatty acid content (*p* > 0.05). By using Differential scanning calorimetry (DSC) analysis, we also found that there was no significant difference in peak temperature (Tp) between the VTR_3_ and EHAR experimental groups (*p* > 0.05). Variable temperature electric heating can affect the flavor of lamb, and there are significant differences in the content of flavor precursors such as fatty acids and amino acids in Xinjiang tannur-roasted mutton.

## 1. Introduction

A famous type of traditional roast mutton is made using a circular clay oven in Xinjiang, China, called a “tannur” by Xinjiang Uyghur people. Burning wood, charcoal, coal, and other fuels in a tannur generates heat radiation to roast mutton. After our investigation, we found that a traditional method of making Xinjiang tannur-roasted mutton involves spraying salt water onto the inner wall of the tannur in the middle stage of roasting to reduce the roasting temperature, and the resulting meat is tender, juicy, and fragrant. Our preliminary research showed that compared to constant-temperature and high-temperature tannur-roasted mutton, variable-temperature roasting can effectively reduce the total content of heterocyclic amines and polycyclic aromatic hydrocarbons. In the sensory evaluation of tannur-roasted mutton, flavor has the highest weight, as compared to taste, tissue morphology, and color [1]. Recently, it was found that stepped sous-vide cooking gives better tenderness, preserves meat color, and significantly reduces cooking loss [2,3]. With the increasing awareness of environmental protection and conservation of nonrenewable energy, charcoal roasting methods are gradually being replaced. Liu et al. stated that electric heating roasting may be a promising alternative to charcoal roasting based on his research on the OAVs and sensory evaluation of nonvolatile compounds and volatile compounds [4]. Xu et al. used the traditional method of burning charcoal to roast oyster cuts of lamb using a core temperature of 79 ± 2 °C for 15, 30, 45, and 60 min. The main volatile compounds or aroma contributors of the oyster cuts were analyzed [5]. Xu et al. used HS-SPME/GC-MS combined with an electronic tongue and electronic nose to evaluate the flavor characteristics and formation of charcoal- and electric-roasted tamarix lamb at different stages of roasting. It was found that electric roasting reduced the content of volatile characteristic compounds, especially aldehydes, alcohols, and ketones [6]. At present, there are few reports on the effects of electric heating and variable-temperature roasting on the characteristic flavor compounds of Xinjiang tannur-roasted mutton.

Lipids are degraded into volatile compounds during food processing, heating, cooking, and storage or interact with other components produced by the Maillard reaction and Strecker degradation, thus contributing to the production of food flavor [7]. Triglyceride (16:0_18:1_18:1) and triglyceride (18:0_18:0_18:1) are the main lipids that affected the stability of aroma binding [8]. A previous study found that vinyl n-caproate, pelargonic acid, and caproic acid were the main volatile compounds when mutton was roasted at 180 °C for 8 min at different rigidity times [9]. 1-Octene-3-ol, derived from lipid β-oxidation, was the most abundant alcohol compound in roast mutton; 1-octene-3-ol is formed via the degradation of linoleic acid, and ketone production is related to the degradation of amino acids and the oxidation of lipids. The main source of aldehydes is lipid oxidation and degradation, and alcohols are mainly derived from lipid oxidation [6,10,11,12]. Aheto et al. found that caproaldehyde can represent the degree of lipid oxidation and flavor formation and can be used as an indicator to evaluate the oxidative stability and flavor acceptability of muscle food [13]. The Strecker degradation of amino acids produces compounds that are aroma contributors in cooked meat [14]. The degradation of sulfur-containing amino acids produces important active intermediates, such as hydrogen sulfide, ammonia, and acetaldehyde, formed by sulfur compounds. Differences in the aroma components of roast mutton with different processing methods or aging times may be mainly caused by water activity and heat transfer efficiency [15]. Exploring the changes in fatty acid and amino acid contents in tannur-roasted mutton under the condition of variable-temperature roasting can effectively reveal the mechanism of flavor formation in tannur-roasted mutton.

Therefore, the aims of this study were to (i) investigate the effects of electric and charcoal-burning roasting at variable temperatures or constant temperatures on the flavor substances of tannur-roasted mutton and (ii) to reveal the correlation between fatty acids, amino acids, and flavor compounds under variable temperatures. The paper provides a theoretical reference and guidance for the production process of variable-temperature tannur-roasted mutton.

## 2. Materials and Methods

### 2.1. Sample Preparation

The samples were obtained from DB 65/T 3788-2015 [16], all sheep had the same genetic background and were fed the same diet. A total of 36 male Duolang sheep (average ketone weight 19.46 ± 2.25 kg for 9-month-olds) were randomly selected and slaughtered according to standard conditions GB/T 43562-2023, [17] and after rigorous maturation, the final pH value was 5.54 ± 0.02. The hindlegs were utilized for analysis and frozen at −20 °C.

**Figure 1:**
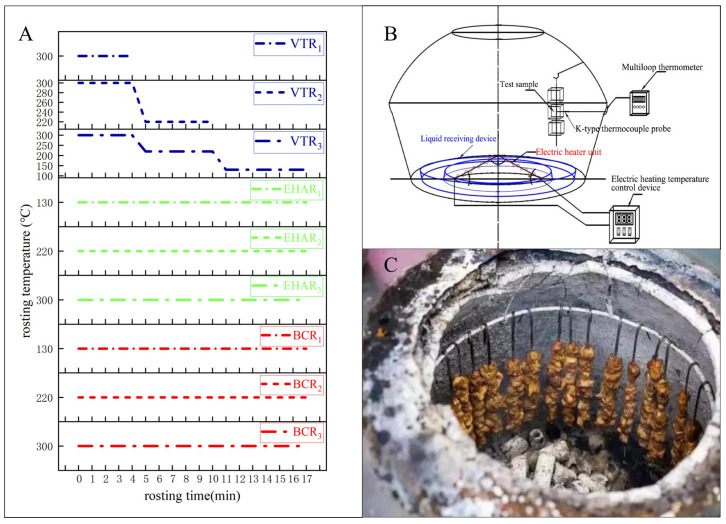
Experimental grouping and roasting conditions. (**A**) Roasting temperature and time of experimental group, VTR: variable-temperature electrically heated air roasting, EHAR: constant-temperature electrically heated air roasting, BCR: constant-burning charcoal roasting; (**B**) a diagrammatic drawing of electrically heated air roasting; (**C**) commercially available and traditional charcoal-burned roast mutton.

The hindlegs were thawed in a low-temperature incubator, and the semitendinosus of the hindlegs was completely removed; the surface fat and muscle membrane were removed, and the semitendinosus was trimmed to 2 cm × 2 cm × 3 cm cubes with the geometric center of the semitendinosus muscle as the center point. Each meat sample weighs approximately 14.3 ± 1.1 g. The muscles from both sides of the carcass were used; the processed semitendinosus cube is considered to have no inter sample differences. We used a completely randomized design method to assign 27 meat samples to 9 experimental groups under VTR, EHAR, and BCR treatments, with 3 meat cubes in each of the treatments. The meat was skewered using stainless steel along the parallel direction of the muscle fibers through the geometric center and then hung in the tannur, which was preheated to the roasting temperature. The electrically heated tannur is equipped with an automatic constant-temperature system, which can keep the error between the setting temperature and the actual temperature within ±1.4 °C. The distance between the meat sample and the electric heating wire is 55.13–70.44 cm, and the distance between the meat sample and the inner wall of the tannur is 10.6–18.3 cm. The sample can be evenly heated during the roasting process. The meat temperature was measured using an infrared thermometer, and the internal temperature of the tannur was measured using a K-type thermocouple probe. In order to avoid systematic errors caused by roasting equipment, we chose the same tannur to ensure the accuracy of the experimental data. Each group of samples is processed separately, and all experimental groups need to pre-heat the tannur to the test temperature before roasting, place the meat samples, and start calculating the time. The experimental group was divided into nine groups. The roasting groups and a schematic diagram of the roasting process are shown in Figure 1. The roasting temperature conditions refer to the optimal variable-temperature roasting conditions in our preliminary research: VTR_1_: electric thermostatic roasting 0–4 min, 300 °C; VTR_2_: electric temperature roasting 0–4 min, 300 °C; 5–10 min, 220 °C; VTR_3_: electrothermal roasting 0–4 min, 300 °C; 5–10 min, 220 °C; 11–17 min, 130 °C; EHAR_1_: electric thermostatic roasting 1–17 min, 130 °C; EHAR_2_: electric thermostatic roasting 1–17 min, 220 °C; EHAR_3_: electric thermostatic roasting 1–17 min, 300 °C; BCR_1_: burning charcoal roast for 1–17 min at 130 °C; BCR_2_: burning charcoal roast for 1–17 min at 220 °C; BCR_3_: burning charcoal roast for 1–17 min at 300 °C; the core temperature and surface temperature of meat samples, respectively, were 81.3–89.6 °C and 84.1–98.4 °C. The meat samples were cooled at 25 °C and 30% humidity for 15 min, vacuum-packed in aluminum foil bags, and stored at −20 °C before analysis. All experiments were performed in triplicate.

### 2.2. Sensory Evaluation

The sensory design was modified from that described by Petričević et al. [18]. A total of 10 sensory panelists were screened and selected based on sensory analysis methodology GB/T 39625-2020 GB/T 13868-2009 [19,20]. Prior to evaluation, all participants were requested to provide informed consent and volunteered to participate. The assessors were generically trained following the procedures of the ISO (11132:2021) standard [21]. All sensory panelists were divided into five groups; each group was composed of one male and one female. The meat slices were cut with a thickness of 0.5 cm in the direction of vertical muscle fibers and placed on white plates. After a group discussion, the six descriptors of the sensory evaluation—fat flavor, grass flavor, roast flavor, smoke flavor, peculiar smell, and acceptability—were selected to access the organoleptic property of the roasted mutton. Each attribute was scored on a 10 cm non-structured line with anchor points at each end (0 = absent; 10 = very strong). The sensory evaluator chewed the sample without swallowing, waited 10 min between the two samples, and gargled with deionized water.

### 2.3. Roasting Losses

Before roasting, the meat surface moisture was dried, and the weight (M_0_) was taken; after roasting, the samples were cooled to room temperature, surface moisture was wiped away, and the weight (M_1_) was taken. The roasting loss rate was calculated as follows:

Roasting losses (%) = (M_0_ − M_1_)/M_0_ × 100 (1)

### 2.4. Differential Scanning Calorimetry (DSC)

The DSC was performed according to the literature [22]. We removed about 3 mm of the surface meat sample, cut it into pieces, weighed and placed 10.0 mg of chopped meat sample in the thermal analysis aluminum crucible after pre-weighing, compacted the meat sample at the bottom of the crucible, and sealed it. An empty aluminum pan was used as a reference, balanced overnight at 4 °C. Measurements were taken using DSC2A-02169 equipment (NETZSCH Geratebau GmbH, Selb/Bavaria, Germany). First, the temperature and sensitivity of the calorimeter were calibrated using indium In (156.6 °C) and a distilled water temperature point (0 °C) with an accuracy of ±0.1 °C. The thermal transition temperature was measured using a DSC single-scan program. The initial equilibrium temperature was 40.00 °C, the temperature rise rate was 1.00 °C/min to 90.00 °C, the nitrogen flow rate in the sample chamber was 20 mL/min, and the protection gas flow rate was 60 mL/min; the DSC thermal phase maps of different meat samples were obtained.

### 2.5. Gas Chromatography–Ion Mobility Spectrometry (GC-IMS)

The volatile compounds were determined using GC-IMS according to the method described by Wang et al. [23]. The tannur-roasted mutton samples were analyzed using GC–IMS (Gesellschaft für analytische Sensorsysteme mbH Corp, Dortmund, Germany) with an FS-SE-54-CB capillary column (15 m × 0.53 mm × 1 μm) (Restek Corp, Bellefonte, PA, USA). The samples were thawed in a refrigerator overnight (12 h) at 4 °C before the experiments.

We used a scalpel to take 3 mm of the surface layer and the internal parts of the VTR, EHAR_2_, and BCR_2_ meat samples, weighing a small amount of each sample. After crushing the meat samples with a meat grinder, we accurately weighed 5.00 g and put it into a 20 mL headspace (HS) vial with a magnetic screw seal cover. The HS incubation temperature was determined to be 55 °C according to the consumer’s roasting and eating temperature; the incubation time was 10 min; the incubation speed was 500 r/min; 1 mL of the HS gas was injected with a syringe into the injector at 60 °C using splitless injection. The cleaning time was 0.50 min. The carrier gas was high-purity nitrogen gas (≥99.999%), and the column was kept at 60 °C. The chromatographic running time was 20 min. The GC column flow rate was set to 2.00 mL/min for 1 min. The rate was increased to 20.00 mL/min for 10 min and 100.00 mL/min for 5 min, and 100.00 mL/min was maintained for 5 min. The samples in the HS injection bottle were incubated, and the HS components in the bottle were extracted with a heated injection needle. The temperature was determined to be 45 °C by IMS.

### 2.6. Free Fatty Acids Analysis

The determination of free fatty acids in the samples was performed according to Yu et al.’s method with some modifications [24]. The 50 mg meat sample was accurately weighed and placed in a 2 mL glass centrifuge tube. After adding a 1 mL chloroform–methanol (2:1) solution, it was treated with ultrasound for 30 min. We removed the supernatant, added 2 mL of 1% methanol sulfate solution, placed it in an 80 °C water bath for 0.5 h, and then added a 1 mL n-hexane solution for extraction, washing with 5 mL pure water. Finally, 25 μL of 500 ppm methyl salicylate was added to 500 mL of supernatant. The extracts were detected using gas chromatography–mass spectrometry (GC-MS). We used capillary columns fitted with DB-WAX (Agilent, Palo Alto, CA, USA). The 7890B-5977B GC-MS instrument (Agilent, with an internal diameter of 30 m × 0.25 mm and a film thickness of 0.25 μm) was used to identify free fatty acids. The sample extract was injected at 280 °C in split mode (1 μL) (10:1). Helium was used as the carrier gas, with a flow rate of 1 mL/min. The column temperature was set at 50 °C for 3 min, then increased by 10 °C/min to 220 °C for 20 min. The mass spectrometer operates in electron-impact (EI) mode at 70 eV, and full-scan mass spectra were collected in the range of 33–1500 *m*/*z*, with the following parameters: temperature of sample inlet: 280 °C; ion source temperature: 230 °C; transmission line temperature: 250 °C. The identification of fatty acids was carried out by comparing the retention time of the FAME peak with the standard.

### 2.7. Free Amino Acids Analysis

The content of free amino acids was determined according to Sabikun et al.’s method with some modifications [25], where 2.0 g of ground meat was weighed and put into a high-temperature test tube, homogenized with 10 mL of distilled water, homogenized with an equal volume of concentrated hydrochloric acid, and charged with nitrogen for protection, before undergoing closed digestion at 110 °C for 21 h, cooling to 25 ± 1 °C, and then filtering using 0.22 μm PVDF. After 100 μL of filtrate was vacuum-dried and redissolved with 1 mL of distilled water, the derivatization treatment was carried out by using ultrasound for 5 min. For the derivation process, we used a mixed standard product, with 50 μL of the sample to be tested plus 50 μL of the protein precipitator (including NVL), mixed at 13,200 r/min for 4 min and refrigerated. We took 10 µL of the supernatant, added 50 µL of labeling buffer to the mix, and performed instantaneous separation. Then, 20 µL of the derivative liquid was added, mixed, and incubated at 55 °C for 15 min. The derived sample was cooled in the refrigerator, mixed, and separated, and 50 µL of the supernatant was taken for detection. We used LC-MS/MS (LC: liquid phase: Dionex UltiMate 3000,Thermo Fisher Scientific Corp, Waltham, MA, USA, MS: mass spectrometry: AB SCIEX API 3200 Q TRAP AB SCIEX Corp, Waltham, MA, USA). The composition of 20 free amino acids—lysine, tryptophan, phenylalanine, methionine, threonine, isoleucine, leucine, valine, serine, glycine, histidine, glutamic acid, glutamine, aspartate, aspartamide, alanine, arginine, proline, cysteine, and tyrosine—in the naan meat was analyzed and tested. For the quantitative amino acid kit scheme MSLAB50AA, we used.(Beijing Mass Spectrometry Medical Research Co., LTD, Beijing, China). The determination was performed using an MSLab50AA-C18 column (150 × 4.6 mm; film thickness: 5 µm) and a column temperature of 50 °C, and the injection volume was 5 μL. The separation was performed at a flow rate of 1 mL/min with the gradient program; mobile phase: aqueous phase (1‰ formic acid), organic phase: acetonitrile (1‰ formic acid), and this was eluted with a gradient as follows: 5% B for 0–1 min; 50% B for 1.1–12 min; 70% B for 12–12.1 min; 100% B for 12.1–15 min; 5% B for 15.1–20 min, respectively. The ESI conditions are as follows: spray voltage: 5500 V; atomizing gas: GS1:55 psi; auxiliary gas: GS2:60 psi; scanning mode: MRM multi-reaction monitoring; atomizing temperature: TEM:500 °C. The free amino acids were identified and quantified by comparing the retention time and peak area of each amino acid standard.

### 2.8. Statistical Analysis

A statistical analysis was performed using the SPSS 20 statistical package (SPSS Inc., Chicago, IL, USA). The significant differences in treatment means were identified at a level of *p* < 0.05 by using a one-way analysis of variance (ANOVA) and the Duncan test. Pearson’s correlations were used for factor analyses. Principal component analysis (PCA) was performed by using OriginPro 2022 (Origin Lab Corp., Northampton, MA, USA).

## 3. Results and Discussion

### 3.1. Effects of Variable-Temperature Roasting Methods on Roasting Loss

**Figure 2:**
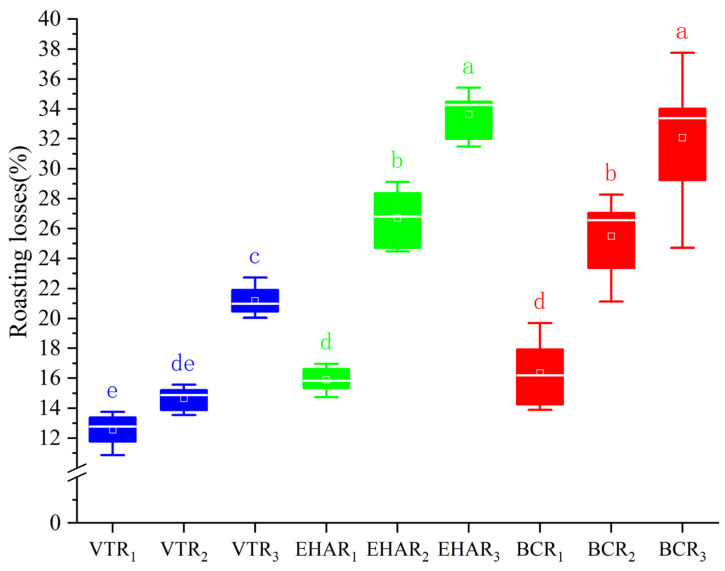
Roasting losses from each experimental group. Different lowercase letters indicate that there is a significant difference (*p* < 0.05). VTR: variable-temperature electrically heated air roasting, EHAR: constant-temperature electrically heated air roasting, BCR: constant-burning charcoal roasting.The white horizontal line in each box plot represents the median, while the small blocks represent the mean value.

Figure 2 indicates the roasting losses of each experimental group. The highest roasting loss in the EHAR_3_ group was 33.65 ± 1.55%, and the roasting losses in the VTR_3_ group were significantly lower than those in the EHAR_2_, EHAR_3_, BCR_2_, and BCR_3_ groups. There was no significant difference in roasting loss between electric- and charcoal-roasted mutton using the same roasting temperature and roasting time (*p* > 0.05).

### 3.2. DSC Thermogram of Tannur-Roasted Mutton

**Figure 3:**
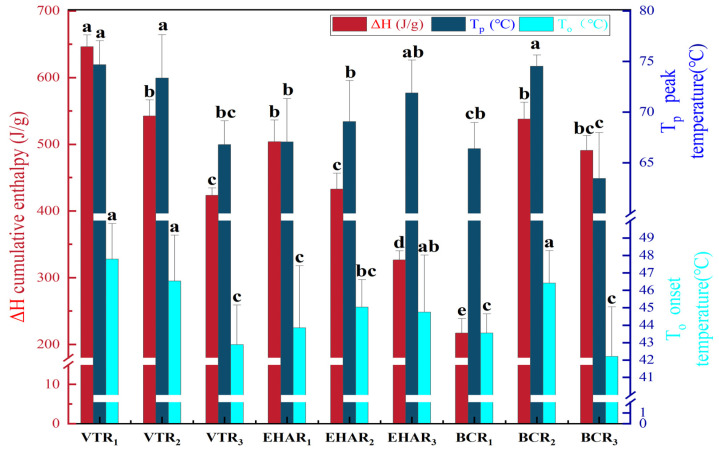
Tp, To, and ΔH from each experimental group. Different lowercase letters in the same row indicate a significant difference (*p* < 0.05). VTR: variable-temperature electrically heated air roasting; EHAR: constant-temperature electrically heated air roasting; BCR: constant-burning charcoal roasting; Tp: The peak temperature; To: onset temperature, ΔH: cumulative enthalpy.

The peak temperature Tp, onset temperature To, and cumulative enthalpy ΔH from the DSC heat flow analysis curve of each experimental group were analyzed, and the results are shown in Figure 3. Tp is the temperature at which the denaturation rate reaches its maximum and represents the temperature of protein denaturation associated with one or more major proteins [26]. According to research findings, a temperature between 61.9 and 66.03 °C in the bovine masseter is the temperature of myosin/endomyosin/perineal collagen denaturation, and the difference in the Tp for beef is due to the different types of myosin [23,27]. In this experiment, the Tp of each roast meat sample ranged from 63.44 °C to 74.67 °C, which was higher than the range of 40.7 °C to 48.7 °C obtained by He et al. for Wuzhumuqin sheep semitendinosus under a heating rate of 10 °C/min [28]. This may be due to the different denaturation of collagen in the intramuscular connective tissue in the roast meat. We also found that there was no significant difference in Tp between the VTR_3_ group and the EHAR experimental groups (*p* > 0.05), which may indicate that the degree of degeneration of the same type of protein was similar between the VTR_3_ group and the EHAR experimental groups. Hwang et al. found that with an increase in temperature, the Tp of pork tenderloin tends to rise to a higher temperature [29], which is consistent with the trend in EHAR_1_–EHAR_3_ in this study in that the higher the roasting temperature, the higher the Tp. Protein denaturation in muscle at Tp is an endothermic process, and the greater the ΔH value [30], the stronger the heat absorption ability of meat. The ΔH value in the VTR_1_ group was significantly higher than that in the other roasting conditions (*p* < 0.05).

### 3.3. Analysis of Volatile Flavor Compounds and Sensory Evaluation

**Figure 4:**
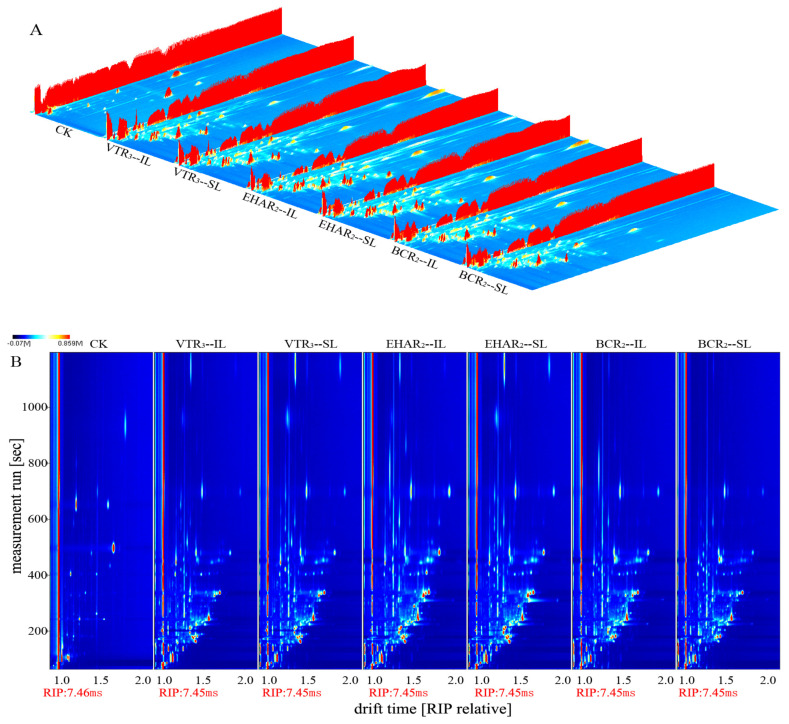
Qualitative analysis of EHAR_2_, VTR_3_, and BCR_2_ characteristic flavor compounds of tannur-roasted mutton using IMS. (**A**) 3D-topographic and (**B**) ion migration spectra of the EHAR_2_, VTR_3_, and BCR_2_ groups. VTR: variable-temperature electrically heated air roasting, EHAR: constant-temperature electrically heated air roasting, BCR: constant-burning charcoal roasting.

Figure 4A shows a 3D-topographic view of the volatile substances in the surface and interior of the meat under three roasting conditions according to the Reporter plug-in in LAV analysis software. In Figure 4B, the horizontal and vertical coordinates represent the gas chromatographic retention time and ion migration time, respectively. Blue is the background color, and the color ranges from white to red with higher concentrations. We selected the best sensory evaluation of variable-temperature roasting under variable-temperature conditions in our preliminary research and used the same roasting time; the experimental groups for a constant temperature of electric heating and burning charcoal roasting at 220 °C, as the usual roasting temperature, were compared and analyzed. It can be seen from Figure 4A/B that volatile substances can be effectively separated using different retention times, and there are differences in the surface and internal volatile substances of the meat samples in the EHAR_2_, VTR_3_, and BCR_2_ groups. By comparing the retention time and drift time using n-ketone C4–C9 as an external standard reference, the retention index of each compound was calculated, and the volatile substances were qualitatively determined by matching them with the GC-IMS library. A total of sixty volatile substances could be clearly characterized, including the monomers and dimers of some substances, with six ketones, fourteen alcohols, thirteen aldehydes, ten esters, three acids, two ethers, five terpenes, four heterocycles, and three other classes. A total of 10 characteristic substances could not be identified in each experimental group (numbers 61–70). The results are shown in Table 1.

**Figure 5:**
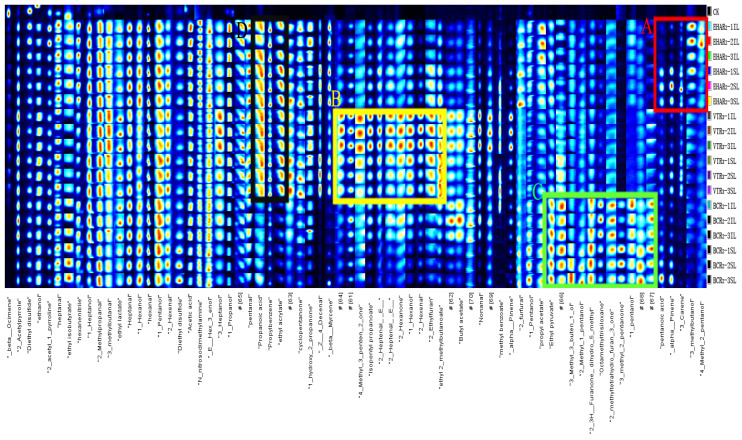
Global overview of the spots identified in the EHAR_2_, VTR_3_, and BCR_2_ group samples at different sampling points. A zone of each topographic plot, which contains most of the important data, is labeled with a rectangle (red, yellow, and green, respectively). IL: internal layer; SL: surface layer.

According to the peak intensity values of 70 characteristic marker substances, the differences in characteristic flavor substances in the samples of the EHAR_2_, VTR_3_, and BCR_2_ groups were analyzed, and their characteristic peaks were obtained, as shown in Figure 5. According to the rectangle region, it can be judged that each sample characteristic flavor has its own characteristic peak region (A–C). Region A (red) is the characteristic peak region of the EHAR_2_ group, including furfural, α-pinene, 3-carene, 3-methylbutanol, 4-methyl-2-amyl alcohol, and five other substances, which are the characteristic volatile substances. Region B (yellow) is the characteristic peak region of the VTR_3_ group, including eleven substances: 4-methyl-3-pentene-2-ketone, isoamyl propionate, trans-2-heptenal, trans-2-heptenal, 2-hexanone, n-hexyl alcohol, 2-hexenal, 2-ethylfuran, 2-methyl-ethyl butyrate, and two characteristic peaks that could not be characterized, which are the characteristic volatile substances. Region C (green) shows the characteristic peaks of the BCR_2_ group, including ethyl pyruvate, 3-methyl-3-butene-1-ol, 2-methyl-1-pentyl alcohol, γ-pentolactone, octamethyltrisiloxane, 2-methyl-tetrahydrofuran-3-one, 3-methyl-2-pentanone, amyl alcohol, and eleven other substances (three characteristic peaks could not be characterized) as the characteristic volatile substances. For region D (black), we compared the BCR_2_ group, and propionic acid, propyl benzene, and ethyl acrylate were the common characteristic volatile substances in the EHAR_2_ and VTR groups. Liu et al. confirmed that hexal, octylaldehyde, 1-octene-3-ol, non-aldehyde, heptal, pentalaldehyde, 3-methylbutyraldehyde, and 2-amylfuran are key aroma compounds in roast mutton [52], and only five volatile substances, including hexal, non-aldehyde, heptal, pentalaldehyde, and 3-methylbutyraldehyde, were detected in this study.

**Figure 6:**
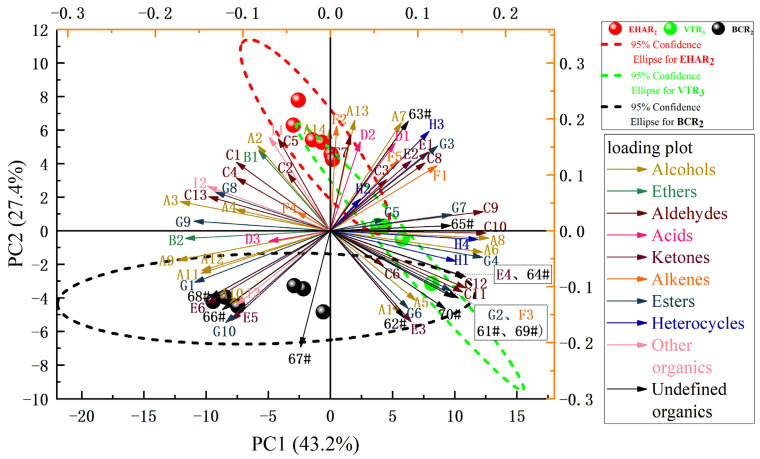
Principal component analysis results of volatile compounds in tannur-roasted mutton. PC1: the first principal component, PC2: the second principal component. Volatiles (loadings) are denoted by their numbers in Table 1. Arrow with line is loading plot of different variance, spherical logo is score plot of principal components.

A principal component analysis was carried out on the characteristic peak retention index values of the flavor substances in each experimental group. Figure 6 shows the principal component score diagram and load diagram of the characteristic flavor substances of each experimental group under the conditions of variable-temperature roasting, electrically heated air roasting, and burning charcoal roasting. The contribution rate of the first principal component (PC1) is 43.2%, the contribution rate of the second principal component (PC2) is 27.4%, and the cumulative contribution rate is 70.6%. This shows that the two principal components can fully represent the main flavor characteristics of the sample. According to the score chart, the closer the distance between samples, the higher the similarity between the aroma components and related contents. The BCR_2_, EHAR_2_, and VTR_3_ groups were distributed in different quadrants and were far away from each other, and the characteristic flavor substances were significantly different. Some regions of the BCR_2_ group and VTR_3_ group overlap within a 95% confidence interval, indicating that the BCR_2_ group and VTR_3_ group may have characteristic flavor substances with similar contributions to PC1 and PC2. According to the load analysis, the substances with the highest positive correlation with PC1 and PC2 were n-hexanol (A8, r = 0.18) and 3-methylbutanol (A13, r = 0.20). In the flavor description, both n-hexanol and 3-methylbutanol had a woody odor.

**Figure 7:**
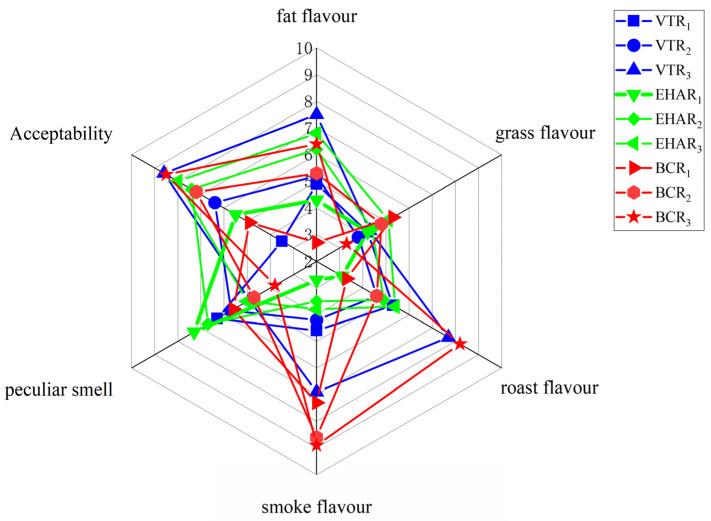
Sensory analysis of the volatile compound profiles from each experimental group. VTR: variable-temperature electrically heated air roasting, EHAR: constant-temperature electrically heated air roasting, BCR: constant-burning charcoal roasting.

As illustrated in Figure 7 the sensory evaluation suggests that each burning charcoal roasting group had a strong smoke flavor, and there was no significant difference between the VTR_3_ group and each charcoal roasting flavor (*p* > 0.05), while the peculiar odor of each EHAR group was stronger than that of each burning charcoal roasting test group. The VTR_3_ group had high acceptability (*p* < 0.05) and a strong roast flavor (7.51 ± 0.27) (*p* < 0.05).

### 3.4. Effects of Variable-Temperature Roasting Methods on Free Fatty Acids

**Figure 8:**
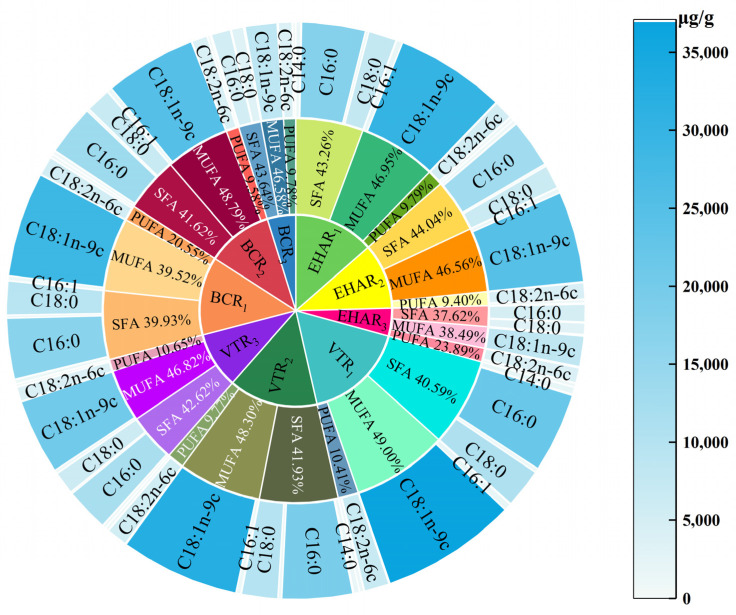
Fatty acids profile from each experimental group. MUFAs: monounsaturated fatty acids; PUFAs: polyunsaturated fatty acids; SFAs: saturated fatty acids. The color intensity, ranging from white to blue, reveals the increase in fatty acid concentration in roasted mutton during the roasting process. VTR: variable-temperature electrically heated air roasting, EHAR: constant-temperature electrically heated air roasting, BCR: constant-burning charcoal roasting. From white to blue, it indicates an increase in content, with darker blue indicating higher content.

Fatty acids store energy in the body, provide energy, and provide a stable cell membrane [53]. Lipids also play an important role in the formation of flavor in meat products. Figure 8 shows the fatty acid content of each experimental group under different roasting conditions. A total of twenty-four fatty acids were detected in this study, including ten saturated fatty acids, six monounsaturated fatty acids, and eight polyunsaturated fatty acids. The content of fatty acids in three fatty groups regarding presence of double bonds in the experimental groups showed the same pattern of ∑MUFA > ∑SFA > ∑PUFA, and the total fatty acid content of the VTR_3_ and BCR_3_ groups had no significant difference (*p* > 0.05), but was lower than that of the other experimental groups (*p* < 0.05). The highest ratios of SFA, MUFA, and PUFA were in the VTR_2_ (44.04%), EHAR_2_ (49.00%), and EHAR_3_ (23.89%) groups. C16:0, C18:1n-9c, and C18:2n-6c had the highest contents among the SFA, MUFA, and PUFA types in the experimental groups. There was no significant difference in the distribution and total content of fatty acids between the electric and charcoal groups at the same roasting temperature and roasting time (*p* > 0.05). This result is similar to the conclusion of Janiszewski et al., where grilling or roasting with the same endpoint temperature had no significant effect on the changes in fatty acid types [54]. Ortuño et al. stated that the heat applied during cooking would promote drip loss of the SFA and MUFA present in adipose tissue [55]. In the present study, we found that the higher the roasting temperature, the lower the total fatty acid content and PUFA/SFA ratio. This is similar to Gerber et al.’s view that different cooking processes reduce total SFA, MUFA, and PUFA contents due to the melting of fats during cooking, thus affecting the composition of fatty acids [56]. In this experiment, it was found that the total content of PUFA/n-6 showed an increasing trend with an increase in roasting temperature, and no change rule was found in the total content of PUFA/n-3 in each group of experiments. When Campo et al. compared various mutton cooking methods (such as braising, roasting, or grilling), no significant difference was found in any single PUFA/n-3 group or in all the PUFA/n-3 groups (*p* > 0.05) [57]. Among the n-3 polyunsaturated fatty acids, only α-linolenic acid (ALA; C18:3 n − 3), eicosapentaenoic acid (EPA; C20:5 n − 3), and docosahexaenoic acid (DHA; C22:6 n − 3) were found. In a study by Corino et al., the most important fatty acids in the most important PUFA/n-3 group were consistent with the three fatty acids found in this experiment [58].

### 3.5. Effects of Variable-Temperature Roasting Methods on Free Amino Acids

**Figure 9:**
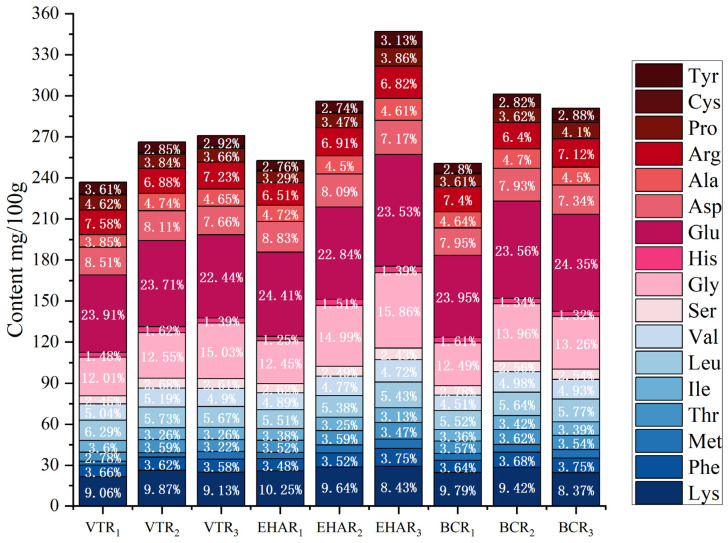
Amino acid analysis from each experimental group. There are seven essential amino acids (EAAs) (Lys, Phe, Met, Thr, Ile, Leu, and Val, represented by the blue series regions in the figure) and ten non-essential amino acids (NEAAs) (Ser, Gly, His, Glu, Asp, Ala, Arg, Pro, Cys, and Tyr, represented by the red series regions in the figure).VTR: variable-temperature electrically heated air roasting, EHAR: constant-temperature electrically heated air roasting, BCR: constant-burning charcoal roasting.

Amino acids play a very important role in the flavor of meat products. They are the precursors of volatile flavor substances in meat products and also affect their taste [59]. The free amino acid content of each experimental group under different roasting conditions is shown in Figure 9, and the columns present the total free amino acid TAA contents of each experimental group. No tryptophan, glutamine, or asparagine was detected in any of the experimental groups. Yujun Xu stated that glutamic acid and alanine are the key amino acids in the taste activity of roast lamb and are the contributors to its taste [4]. Glutamic acid had the highest content among the free amino acids in all the experimental groups, and the content of glutamic acid in VTR_3_ was the lowest at 22.44%. The total free amino acid content (347.05 mg/100 g) in the EHAR_3_ group was significantly higher than that in the other experimental groups (*p* < 0.05), but the proportion of essential amino acids (EAA/TAA) was 30.88%, which was lower than that in the other experimental groups. Elmore et al. stated that the Maillard reaction degrades cysteine to form an important flavor substance in mutton [60]. In this study, the content of cysteine was the lowest among the amino acids, at 0.32–0.68%.

### 3.6. Correlation between Roasting Loss, Dsc, Free Fatty Acids, and Free Amino Acids

**Figure 10:**
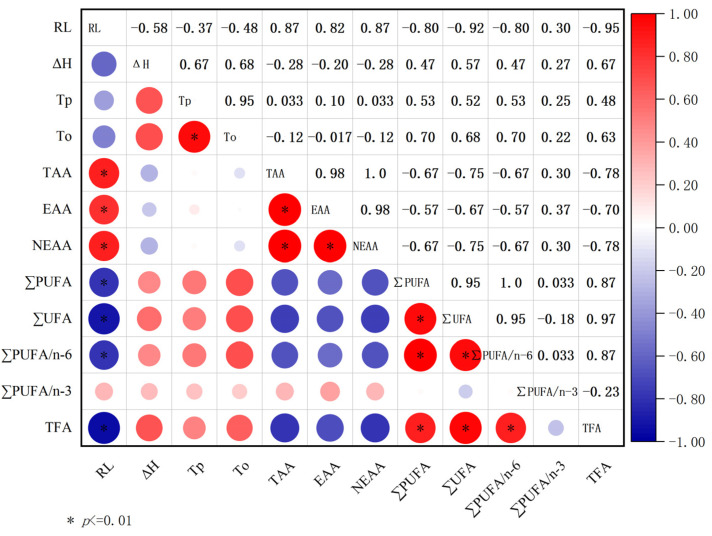
Correlation analysis for roasting losses, DSC, free fatty acids, and free amino acids. The red unit indicates a positive correlation, where the correlation is greater than the red unit. Similarly, the blue unit is negatively correlated, and the blue unit is more correlated. RL: roasting losses; Tp: the peak temperature; To: onset temperature; ΔH: cumulative enthalpy; TAA: total free amino acid; EAA: essential amino acid; NEAA: non-essential amino acid; MUFA: monounsaturated fatty acid; PUFA: polyunsaturated fatty acid; SFA: saturated fatty acid; TFA total fatty acid.

Figure 10 shows the Spearman correlation analysis results for the roasting loss, DSC analysis, fatty acids, and free amino acid indexes of each experimental group. The roasting loss was significantly negatively correlated with EAA, NEAA, and TAA (the correlation coefficients (r) were 0.82, 0.87, and 0.87, respectively), with *p* < 0.01. There was no significant correlation between EAA, NEAA, and TAA contents and ∑MUFA, ∑PUFA, PUFA/n-6, and PUFA/n-3, and there was no significant correlation between Tp, To, ΔH, and free amino acids and fatty acids (*p* > 0.05).

## 4. Conclusions

In summary, the volatile components and characteristic flavor of Xinjiang tannur-roasted mutton obtained using variable-temperature roasting, electrically heated air roasting, and burning charcoal roasting were compared and analyzed. Compared with the EHAR_2_, EHAR_3_, BCR_2_, and BCR_3_ groups, the high-temperature roasting time in the VTR_3_ group was shortened, and the roasting loss was significantly reduced. Variable temperature roasting and charcoal roasting have the same number of characteristic flavor compounds, while constant-temperature electric heating roasting has the least characteristic flavor compounds, only six, and the constant-temperature electric heating roasting has a poorer flavor. We believe that n-hexanol (A8, r = 0.18) and 3-methylbutanol (A13, r = 0.20) had a relationship and affect the aroma. A sensory analysis showed that VTR_3_ had high acceptability and fat flavor. The total fatty acid content and PUFA/SFA ratio decreased with a higher roasting temperature. Roasting loss was negatively correlated with EAA, NEAA, and TAA, but there was no correlation between the free amino acid content and fatty acid content. The variable-temperature electrically heated air roasting method was associated with different characteristic aroma substances in tannur-roasted mutton when compared to constant-temperature electric heating roasting and burning charcoal roasting. We found that there were significant differences in the characteristics of flavor compounds, fatty acids, amino acids, and roasting losses between the variable temperature roasting and constant-temperature electric heating roasting experimental groups. We inferred that this may be due to the influence of variable-temperature roasting on organic substances such as acids and amino acids, which may promote the occurrence of the Maillard reaction and Strecker degradation, among other component interactions. The variable-temperature electrically heated air roasting condition had better acceptability in the sensory evaluation. This study provides scientific data on the characteristic aroma substances of mutton roasted in a tannur with variable-temperature electrically heated air, providing a theoretical basis for production technology.

## Figures and Tables

**Table 1:** Gas chromatography–ion mobility spectrometry (GC-IMS) integration parameters of volatile compounds in tannur-roasted mutton.

Volatiles	Serial Number	Compound	Flavor Description	Characteristic Peak Number	CAS#	RI	Rt(s)	Dt (ms)	References
Alcohols	A1	Ethanol	/	4	C64175	487.1	86.96	1.0505	
	A2	1-Heptanol	green, musty, pungent	9	C111706	968.7	484.533	1.4068	[31]
	A3	1-Hexanol-D	woody, cut grass, chemical–winey, fatty, fruity, weak metallic	41	C111273	877.8	327.663	1.6299	[32]
	A4	1-Pentanol-M	roasted	63	C71410	778.4	222.813	1.5179	[33]
	A5	(E)-Hex-3-enol	/	21	C928972	830	272.228	1.2607	
	A6	3-Heptanol	fresh, grassy, fatty, rancid	22	C589822	884.1	335.798	1.6625	[34]
	A7	1-Propanol	/	23	C71238	541.8	101.915	1.1163	
	A8	1-Hexanol-M	woody, cut grass, chemical–winey, fatty, fruity, weak metallic	14	C111273	863.9	310.489	1.3263	
	A9	1-Pentanol-D	roasted	16	C71410	785.4	229.037	1.2543	
	A10	3-Methyl-3-buten-1-ol	sweet fruity	57	C763326	737.2	189.156	1.1598	[35]
	A11	2-Methyl-1-pentanol	caramel	58	C105306	845	288.529	1.3005	[36]
	A12	1-Pentanol-D	roasted	53	C71410	766.5	212.484	1.2549	
	A13	3-Methylbutanol	woody, acorn, pleasant green	69	C123513	738.7	190.334	1.4782	[37]
	A14	4-Methyl-2-pentanol	/	70	C108112	764.8	211.091	1.2882	
Ethers	B1	Diethyl disulfide-M	moldy, sulfur	3	C110816	926.2	402.129	1.1526	[38]
	B2	Diethyl disulfide-D	moldy, sulfur	18	C110816	926.2	402.129	1.287	[38]
Aldehydes	C1	Heptanal-M	fatty, putty	6	C111717	891	344.837	1.3342	[39]
	C2	2-Methylpropanal	fresh, aldehydic, floral, pungent	10	C78842	563.1	108.396	1.0887	[40]
	C3	3-Methylbutanal	caramel, chocolate	11	C590863	647.1	138.307	1.1577	[41]
	C4	Heptanal-D	fatty, putty	13	C111717	886.9	339.413	1.6977	
	C5	Hexanal	fruity, fatty, green	15	C66251	804.1	246.314	1.5661	[42]
	C6	2-Hexenal-M	salty meat, dry ham	17	C505577	846.6	290.355	1.1842	[33]
	C7	Pentanal	salty, musty odors	25	C110623	689.8	156.752	1.1789	[43]
	C8	(Z)-4-Decenal	fruity, heavy, sweet	32	C21662099	1201.3	1142.345	1.3425	[44]
	C9	(E)-2-Heptenal-M	fatty, fruity	38	C18829555	955.6	457.538	1.2534	[45]
	C10	(E)-2-Heptenal-D	fatty, fruity	39	C18829555	954.2	454.697	1.6843	[45]
	C11	2-Hexenal-D	salty meat, dry ham	42	C505577	845.3	288.859	1.5154	
	C12	Nonanal	citrus, green, citronella grass	48	C124196	1064.1	697.868	1.9507	[46]
	C13	2-Furfural	almond, woody	52	C98011	828.8	271.033	1.3369	[44]
Acids	D1	Acetic acid	pungent, acidic, cheesy, vinegar	19	C64197	643.4	136.812	1.0675	[47]
	D2	Propanoic acid	/	26	C79094	696.9	161.239	1.2638	
	D3	Pentanoic acid	sweaty	66	C109524	902.5	362.42	1.2203	[44]
Ketones	E1	Cyclopentanone	/	30	C120923	767.8	213.583	1.3296	
	E2	1-Hydroxy-2-propanone	butter, sweet	31	C116096	652	140.301	1.25	[44]
	E3	4-Methyl-3-penten-2-one	sweet, chemical	36	C141797	792.7	235.604	1.4431	[48]
	E4	2-Hexanone	/	40	C591786	790.1	233.242	1.4895	[48]
	E5	2-Methyltetrahydro-furan-3-one	caramel, green	61	C3188009	794	236.865	1.4242	[36]
	E6	3-Methyl-2-pentanone	/	62	C565617	756.6	204.357	1.4817	
Alkenes	F1	Beta-Ocimene	/	1	C13877913	1044.6	650.762	1.2183	
	F2	Beta-Myrcene	green	33	C123353	984.2	518.631	1.2987	[41]
	F3	Alpha-Pinene-M	sharp, pine	67	C80568	930.4	409.598	1.6718	
	F4	Alpha-Pinene-D	sharp, pine	51	C80568	935.9	419.76	1.2189	
	F5	3-Carene	earthy, green, fresh	68	C13466789	1007	568.552	1.2189	[42]
Esters	G1	Ethyl isobutyrate	/	7	C97621	734.5	187.162	1.1906	
	G2	Ethyl lactate	/	12	C97643	798.4	240.891	1.527	
	G3	Ethyl acrylate	/	27	C140885	703.1	165.227	1.3997	
	G4	Isopentyl propanoate	/	37	C105680	967.3	481.691	1.8275	
	G5	Ethyl 2-methylbutanoate	fruity, sweet	44	C7452791	864.2	310.818	1.6465	[44]
	G6	Butyl acetate	fruity	46	C123864	824	266.014	1.6138	[49]
	G7	Methyl benzoate	/	50	C93583	1095.1	780.014	1.216	
	G8	Propyl acetate	/	54	C109604	708.3	168.683	1.1636	
	G9	Ethyl pyruvate	/	55	C617356	727	181.678	1.422	
	G10	2(3H)-Furanone, dihydro-5-methyl	cardboard and metallic	59	C108292	954.2	454.697	1.417	[50]
Heterocycles	H1	2-Acetylpyrrole	toasted	2	C1072839	1066.3	703.33	1.4798	[51]
	H2	2-Acetyl-1-pyrroline	green, strange, coffee, sweet, fried corn	5	C85213225	927.5	404.493	1.4723	[51]
	H3	Propylbenzene	/	27	C103651	948.4	443.331	1.1599	
	H4	2-Ethylfuran	fruity, floral	43	C3208160	698.1	161.991	1.3142	[39]
Other organics	I1	hexanenitrile	/	8	C628739	875.6	324.951	1.2625	
	I2	N-nitrosodimethylamine	/	20	C62759	747.6	197.132	1.0632	
	I3	Octamethyltrisiloxane	/	60	C107517	877.1	326.847	1.5509	

RI: retention index, calculated using n-ketones C4–C9; Dt: drift time in the drift tube; Rt: retention time in the capillary GC column; D: dimer; M: monomer.

## Data Availability

The original contributions presented in the study are included in the article, further inquiries can be directed to the corresponding author.
